# Shared decision-making between paediatric haematologists, children with sickle cell disease and their parents: an exploratory study

**DOI:** 10.1007/s00431-023-05280-x

**Published:** 2023-10-31

**Authors:** Ricardo Wijngaarde, Mijra Koning, Karin Fijnvandraat, Dirk Ubbink

**Affiliations:** 1 Department of Surgery, Amsterdam University Medical Centers, University of Amsterdam, Meibergdreef 9, 1105 AZ Amsterdam, The Netherlands; 2 Emma Children’s Hospital, Amsterdam University Medical Centers, AMC, Meibergdreef 9, 1105 AZ Amsterdam, The Netherlands; 3 Alkmaar Medical Center, Department of Internal Medicine, Noordwest Ziekenhuisgroep, Wilhelminalaan 12, 1816 JD Alkmaar, The Netherlands; 4 Department of Pediatric Hematology, Amsterdam University Medical Centers, location University of Amsterdam, Meibergdreef 9, 1105 AZ Amsterdam, The Netherlands

**Keywords:** Shared decision-making, Paediatrics, Sickle cell disease, Triadic decision-making, Risk communication, Chronic disease

## Abstract

Children with sickle cell disease (SCD) face various healthcare choices to be made during the disease process that may impact their lives. Shared decision-making (SDM) could improve their health outcomes. We assessed if, and to what extent, paediatricians engage children with SCD and/or their parents in the decision-making process. In this observational cross-sectional study, paediatric SCD patients and their parents visiting the outpatient paediatrics clinic of a university hospital participated in a SDM baseline measurement. Two evaluators independently and objectively analysed the level of patient involvement in decision-making from the audio-recordings of the consultations using the OPTION-5 instrument, a 0–20-point scale from which scores are usually expressed as a percentage of ideal SDM. The level of SDM, as perceived by patients, parents and paediatricians, was appreciated using the SDM-Q-9 and SDM-Q-Doc questionnaires, respectively. Scores could range from 0% (no SDM) to 100% (exemplary SDM). Twenty-four consultations in which a decision needed to be made about SCD treatment were audiotaped and analysed; six were from each paediatrician. The group consisted of 17 male and 7 female patients from various cultural backgrounds between 2 and 17 years old, with a mean age of 9.4 years (SD 4.2). Median OPTION-5 scores were 25.0% [IQR] 20.0–40.0%; range 0–55%). Median SDM-Q-9 and SDM-Q-Doc scores were 56.7% (IQR 39.4–88.9%) and 68.9% (IQR 57.8–77.8%), respectively.

*Conclusion*: Although subjective scores of SDM were fair, the objectively scored level of SDM among children suffering from SCD leaves room for improvement. This may be realized by increasing knowledge about the benefits of SDM, child-centred SDM interventions and SDM-training for paediatricians that takes into account the complexity of intercultural challenges and risk communication between stakeholders.

**Table:** 

**What is Known:** *• Children that suffer from sickle cell disease (SCD) are more vulnerable to factors that negatively impact the care that they receive as well as suboptimal health outcomes.* *• Shared decision-making (SDM) can help children participate in a collaborative decision-making process about their preferred treatment options and improve their health outcomes.*	
**What is New:** *• The level of participation in the decision-making process for patients suffering from SCD and the families that they belong to leaves room for improvement. The impact of intercultural challenges and the quality and consistency of risk-communication between stakeholders in paediatric SDM needs further exploration.* *• Paediatricians are more confident about their ability to involve the child and parents compared to how children and their parents experience their level of involvement in a shared decision-making process.*

**What is Known:**

*• Children that suffer from sickle cell disease (SCD) are more vulnerable to factors that negatively impact the care that they receive as well as suboptimal health outcomes.*

*• Shared decision-making (SDM) can help children participate in a collaborative decision-making process about their preferred treatment options and improve their health outcomes.*

**What is New:**

*• The level of participation in the decision-making process for patients suffering from SCD and the families that they belong to leaves room for improvement. The impact of intercultural challenges and the quality and consistency of risk-communication between stakeholders in paediatric SDM needs further exploration.*

*• Paediatricians are more confident about their ability to involve the child and parents compared to how children and their parents experience their level of involvement in a shared decision-making process.*

## Introduction

From a human rights perspective, children have a moral and legal right to be involved in decision-making regarding their own health (CRC). Within healthcare, shared decision-making (SDM) has become an essential and ethically sound collaborative decision-making standard. SDM may also enhance a child’s decision-making involvement because it incorporates patients’ preferences about treatment options to foster better health outcomes. SDM among adults has shown to enhance treatment adherence, reduce decisional conflict and increase patient satisfaction [1, 2]. SDM in paediatrics shows similar results [3, 4].

Sickle cell disease (SCD) is a serious and life-limiting congenital medical condition due to a defect of the beta-haemoglobin gene that mostly affects populations from South Asian, Middle Eastern, sub-Saharan African and Mediterranean areas. Symptoms include vascular occlusion, organ failure and pain episodes. SCD negatively impacts a child’s quality of life and increases morbidity and mortality. During the course of this disease, several treatment options may be discussed, depending on the situation and preference of the child and their parents. For this reason, the involvement of these stakeholders through SDM seems a viable approach to arrive at the healthcare decisions that best fit their situation and preferences.

In general, to apply SDM in clinical practice, both clinicians and patients need to be aware of the principles of SDM. In paediatrics, SDM may be more challenging. An important distinguishing characteristic is the involvement of parents whose legal authority to decide for the child needs to be balanced against a child’s growing decisional capacities [5–7]. This implies a triadic shared decision-making process in the child’s best interest that involves child, parent(s) and physician [8–11]. In case of chronic diseases in children, the transition from a dyadic (parent-physician; when the child is young) to a triadic (child-physician-parent; when the child grows up) and finally back to a dyadic (nearly grown-up adolescent — physician) decision-making process can affect the course and outcome of a collaborative SDM process between different stakeholders [12–14].

Since SDM requires the opportunity of weighing risks and benefits from different treatment options, risk communication is an essential, integral part of SDM [12]. Studies show that maintaining the quality and consistency of risk communication can be more challenging when a chronically ill child’s best interests and parent-physician intercultural challenges are not addressed [15, 16]. The quality and consistency of this information exchange also affects the quality and outcome of a decision-making process on behalf of children [17–20]. Apart from communication and language barriers, actual or perceived health illiteracy among parents also impacts their level of understanding of the information provided about treatment options, risks, and benefits. Given these challenges, as a starting point to improve the level of SDM, we investigated the current level of SDM with children suffering from SCD during clinical visits to their paediatric haematologist.

## Methods

This observational, cross-sectional study was conducted as a baseline assessment of the level of SDM at the Paediatric Haematology department of the Amsterdam University Medical Centers (UMC), specialized in SCD. The full team of four paediatric haematologists participated, none of whom had received previous SDM training. Consultations with the nursing staff were not included as they were not involved in treatment decision-making. This study was reported along the STROBE guidelines (Von Elm). The Amsterdam UMC medical ethics review board waived the need for a full review (Registration number W21_312 # 21.347).

### Patients

(Parents of) patients suffering from SCD and visiting the outpatient Paediatrics clinic were eligible for this study. Inclusion criteria were:

- Children suffering from SCD
- Age range 0–18 years
- Need for a health-related decision

Excluded were those with haematologic diseases other than SCD, as were patients who did not consent to participate, even if their parents agreed to participate.

### Questionnaires and SDM measures 

The Observing Patient Involvement (OPTION-5) instrument is a validated tool to measure the level of patient involvement in clinical decision-making (Barr) by an independent researcher, using audio-recordings of the consultations. The OPTION-5-item is derived from the original OPTION-12-item instrument [2] and has proven to be less burdensome for the observer as compared to the OPTION-12, while maintaining strong psychometric properties [21–23]. The instrument addresses five items that are considered essential elements of a shared decision-making process (see Table 1). Item scores range from 0 (not observed) to 4 (exemplary effort) regarding the application of SDM (Fig. 2). Total OPTION-5 scores can range from 0 to 20.

**Table 1:**
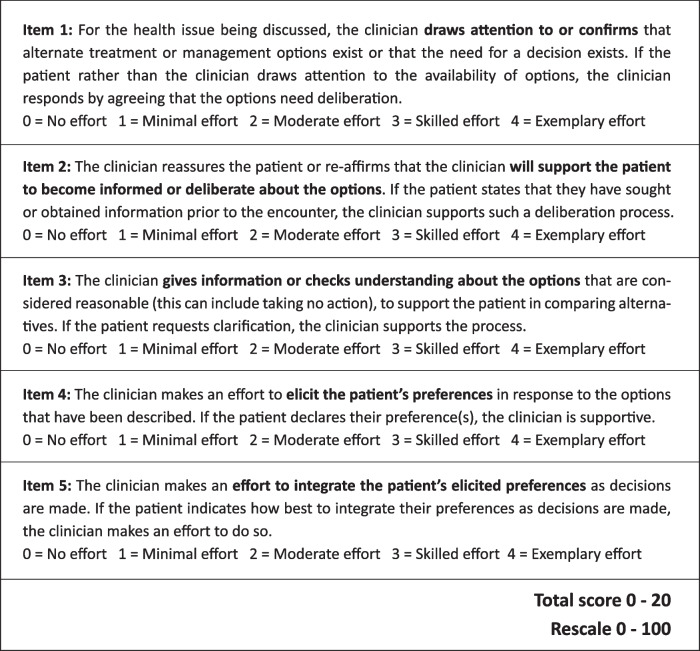
Option-5 instrument

The validated SDM-Q-9 and SDM-Q-Doc questionnaires measure the levels of involvement in SDM as perceived by patient and physician, respectively. Unlike the OPTION-5 instrument, the SDM-Q-9 and the SDM-Q-DOC measure the level of involvement from a subjective perspective. In this study, the Dutch versions of the SDM-Q-9 and the SDM-Q-Doc questionnaires were used [24]. These nine-item scales contain 5-point Likert questions, which can add up to a maximum score of 45 (Tables 2 and 3).

**Table 2:**
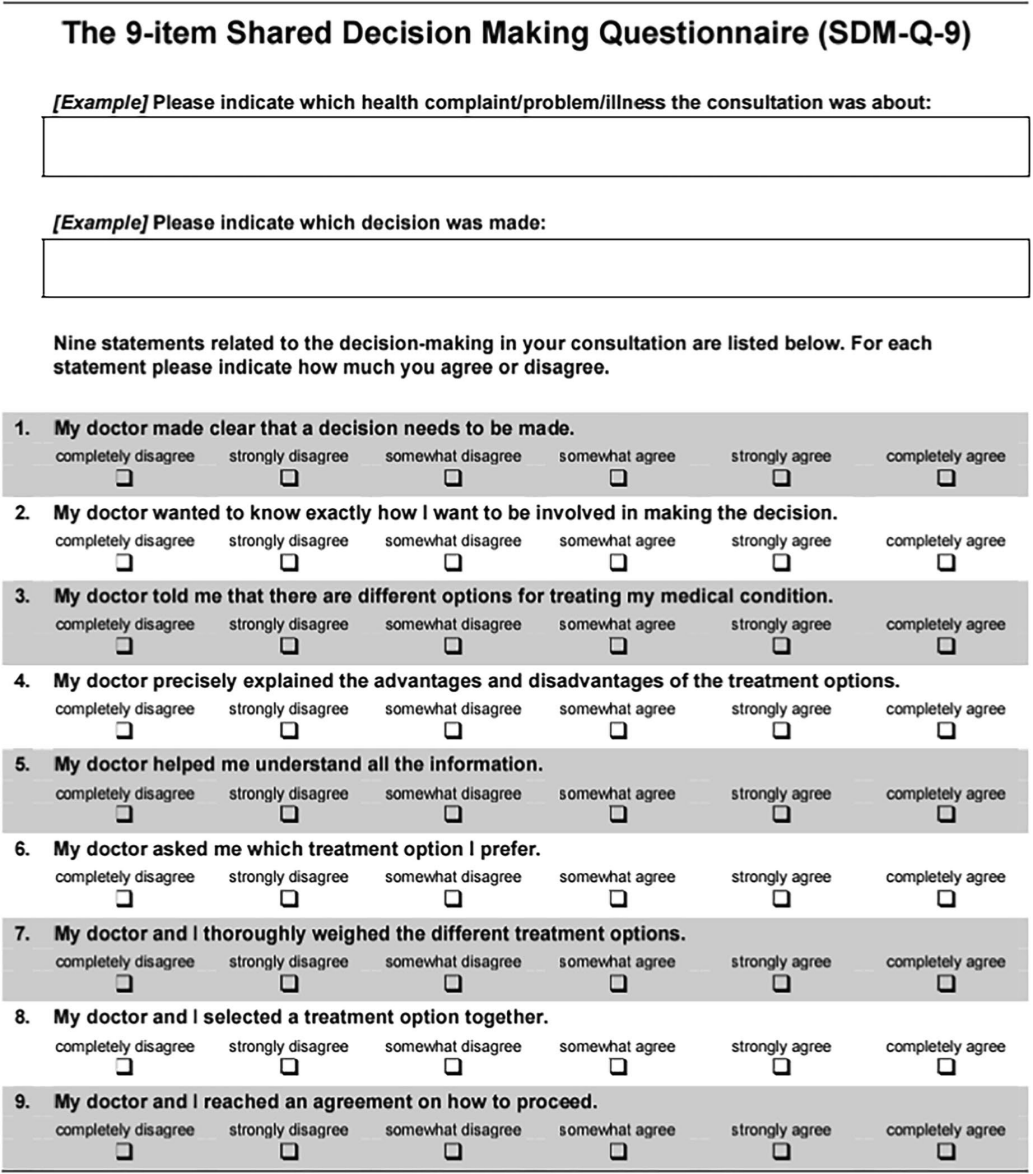
SDM-Q-9 questionnaire

**Table 3:**
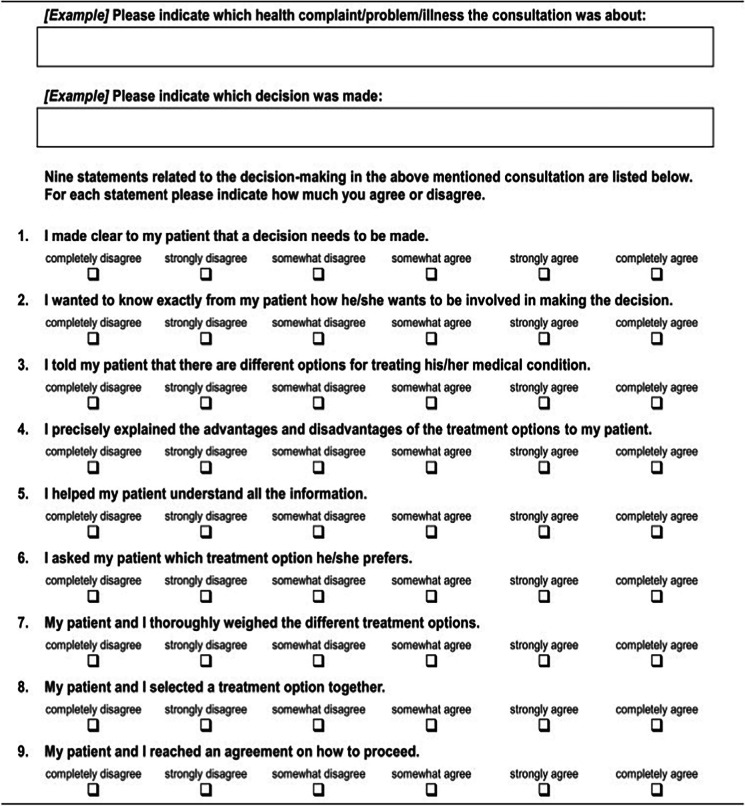
SDM-Q-Doc questionnaire

### Study conduct

From September 2021 to January 2022, patients with SCD visiting the outpatient Paediatrics clinic were invited to participate. After having obtained informed consent, basic demographic data such as age, sex and duration of the disease) were collected before the consultation. The consultations, either face-to-face or by telephone, between paediatrician and (parents of) patients were audio-recorded. At least five consultations per paediatrician were to be collected to obtain sufficiently reliable individual SDM scores [25].

Immediately after the consultation, patients or their parents — if children were below the age of twelve — were asked to fill in the SDM-Q-9, and paediatricians were asked to complete the SDM-Q-Doc questionnaire and to provide some demographics (age, sex, years of experience).

### Statistical analysis

Statistical analysis was performed using SPSS (IBM SPSS v. 28, Armonk, NY, USA). Overall SDM and OPTION scores were transformed into percentages of the maximum score (0% meaning no SDM observed; 100% indicates exemplary SDM). Descriptive statistical analysis was executed by calculating mean values of the questionnaire scores with their 95% confidence intervals or the medians with interquartile ranges (IQR) if not evenly distributed. To determine the OPTION-5 scores accurately and objectively, two evaluators (RW and MK) separately rated the first series of six audiotaped consultations, after which the separate scores were compared using the OPTION guidelines and measurement interpretation guide [26]. OPTION-5 scores were calculated for each item separately to assess inter-observer agreement by calculating the kappa value. This process was repeated until a kappa of > 0.6 (equalling substantial agreement) was reached, after which the remaining recordings were analysed by a single evaluator. A Bland–Altman plot was used to appreciate the level of agreement between the two questionnaires and whether the differences were systematic and constant over the full range of scores [27]. A related samples Wilcoxon Signed-Ranked test was performed to measure the significance in differences between the SDM-Q-9 and SDM-Q-DOC scores.

## Results

**Fig. 1:**
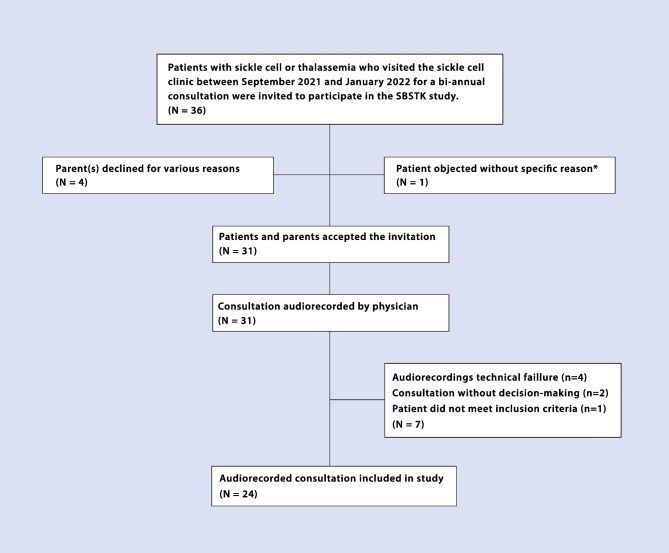
Flowchart of consultation inclusion

Between September 2021 and January 2022, 31 consultations were recorded, of which 24 (six per paediatrician) were used in our study. Seven audio-consultations were excluded for the following reasons: technical malfunction that made the recording partly or entirely inaudible (*n* = 4), patient was suffering from another blood disease than SCD (*n* = 1) or no health-related decisions were made (*n* = 2). Two consultations were conducted by phone, one of which was included in our study. Study flowchart and SDM stakeholder characteristics are shown in Fig. 1 and Table 4, respectively. Our patient population had a mean age of 9.4 years (range 2–17 years, SD 4.2) with 17 out of 24 (71%) patients being male. Median duration of the consultations was 27:43 (mm:ss; IQR 14:32), ranging from 11:49 to 01:23:03 min (Table 4). The duration of the remote consultation was 16:23 min. No patient decision-making support tools were used before or during the consultations.

**Table 4:** Participant data

**Patients**		**Gender**		**Age**			
	***N***	Female		Min	Max	Mean	SD
	24	7		2	17	9.4	4.2

**Parents/caregivers**		**Biological**					
	***N***	Yes	No				
	22^a^	19	3				

**Physicians**		**Gender**		**Experience as paediatrician (yrs)**			
	***N***	Female		Min	Max	Mean	SD
	4	4		8	24	15	6.8

				**Experience as Haematologist (yrs)**			
				Min	Max	Mean	SD
				3	22	11.5	7.8

**Audiorecordings**		**Location**		**Duration (minutes:seconds)**			
	***N***	In clinic	Teleconsult	Min	Max	Median	SD
	24	23	1	11:49	38:42	27:43^b^	08:19

^a^Two teenagers visited the outpatient clinic unaccompanied

^b^One conversation with patient and parents living overseas lasted 01:23:03. When this conversation was not included in the calculation, the mean duration of the audio recordings was 25:09

### OPTION-5 scores

**Fig. 2:**
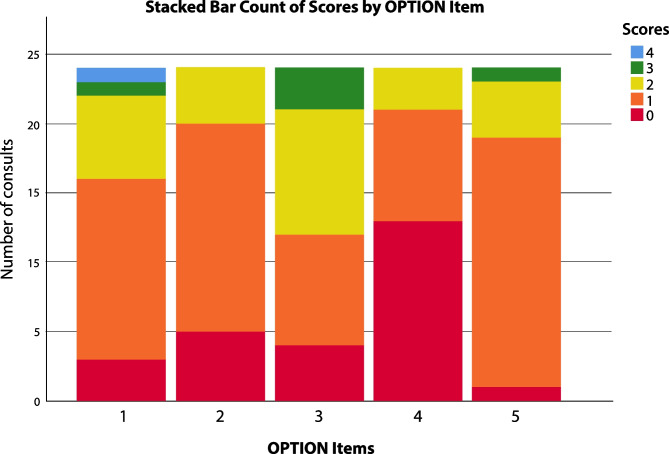
Total option scores per item. *OPTION-5 scores for each of the 5 items: 0, not observed; 1, baseline level; 2, moderate level; 3, skilled level; 4, exemplary level

Interrater reliability scored a *kappa* value of 0.71 after measuring two consecutive sets of 6 conversations. Mean total OPTION-5 score was 27.7% (SD 13.8%) with a median score of 25% (IQR 20–40%, range 0–55%). As shown in Fig. 2, item 3 of the OPTION-5 instrument (‘Assuring the patient that he/she will inform and help the patient make understand and weigh the proposed treatment options’) was observed most frequently. Item 4 (‘Exploring patients’ expectations and discussing the patients’ preferences and concerns’) was least observed. The analysis of the audio-recordings showed that all paediatricians made a deliberate effort to explain the pros and cons of only one treatment option, rather than discussing any alternative options as well. For example, pneumococcal vaccination is an important and frequently presented prophylactic treatment for SCD patients. Typically, this vaccination was recommended without gauging the patient’s preference regarding vaccinations. In addition, information exchange about treatment options was not followed by a ‘teach back’ action, which also limited the scores. Other OPTION-5 items that received low scores addressed the paediatrician’s reassurance of the patient/parent that he/she will be sufficiently informed so as to participate in the decision-making process (item 2) as well as taking patient/parents preferences into account in the final decision-making (item 5).

### SDM-Q-9 and SDM-Q-Doc scores

**Fig. 3:**
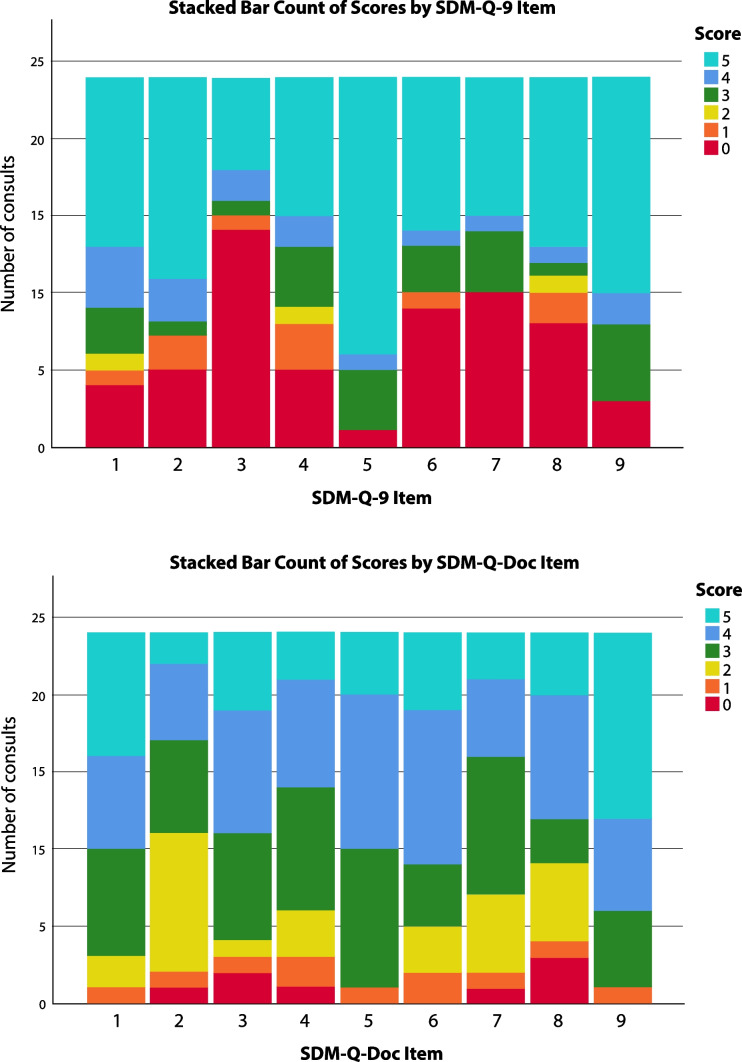
SDM-Q-9 and SDM-Q-Doc total scores per item, respectively. 1, Clarifying that a decision needs to be made. 2, Eliciting patients involvement preferences. 3, Clarifying multiple ways to deal with the health-related problem. 4, Explaining the pros and cons of each (non)treatment option. 5, Patients level of understanding of the provided information. 6, Identifying patients preferred (non)treatment option. 7, Weighing of the discussed (non)treatment options. 8, Choosing a treatment option together. 9, Shared agreement on follow-up arrangements

**Fig. 4:**
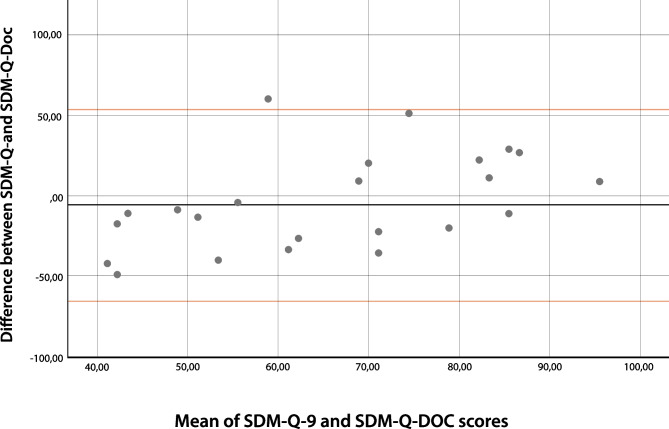
Bland–Altman plot of the differences between SDM-Q-9 and SDM-D-doc against the mean values. The black horizontal line indicates the mean difference between the Q-9 and the Q-Doc scores. The red lines indicate the 95% limits of agreement between Q-9 and the Q-Doc scores

The SDM-Q-9 scores (range 17.8–100%) showed a wider range than the SDM-Q-Doc scores (range 28.9–91.1%) (Fig. 3). Overall, SDM-Q-9 scores were not significantly lower than the SDM-Q-Doc scores (*p* = 0.277). The B&A plot (Fig. 4) showed that individual SDM-Q-9 scores were not systematically lower than the SDM-Q-Doc scores (mean difference − 5.8%, with wide 95% limits of agreement (− 65.4 to + 53.8). The B&A plot also showed a trend towards a higher difference in SDM-Q scores (Q-9 minus Q-Doc) with increasing mean scores. The patients’ median SDM Q-9 score was 56.7% (IQR 39.4–88.9%), while 3 out of 24 (12.5%) of them gave the maximum score (100%) (see Fig. 3). The SDM-Q-9 item that scored highest was item 5 (‘Investigating if the patient has understood the information’), whereas item 3 (‘Stating that there is more than one way to deal with the health-related problem’) scored the lowest. Patients/parents felt that the paediatrician made a deliberate effort to elicit their preferred level of involvement (item 2). The same was true for how they experienced paediatricians’ effort to determine if they had understood the information provided (item 5).Two SDM-Q-9 items indicated that patients/parents felt less involved by the paediatrician in the decision-making process. One item addressed whether patient/parents’ preferred treatment option was taken into account (item 6) while the other (item 7) addressed whether they were involved by the paediatrician in the process of weighing the pros and cons of different treatment options. The paediatricians’ median SDM-Q-Doc score was 68.9% (IQR 57.8–77.8%) (Fig. 3). Item 9 (‘Agreement on follow-up arrangements’) scored highest, while item 2 (‘Eliciting the patients preferred involvement in the decision-making’) scored the lowest. Paediatricians considered themselves skilled when clarifying that a health-related decision needed to be made (item 1) and when making the patient/parent aware of multiple treatment options (item 3). Paediatricians were not confident about their own efforts to explain and then weigh the pros and cons of different treatment options as addressed by items 4 and 7, respectively.

## Discussion

Although parents of patients with sickle cell disease expressed satisfaction with the care they received from their team of haematology specialists, the objectively observed level of SDM shows room for improvement. This was also observed in previous studies in other areas of medicine [28]*.* In our study, this may be due to a (possibly unconscious) treatment preference among paediatricians, which may have led to discussing only one option, and thereby nudging the parent/patient to choose this option [29, 30]. Audio-recordings indicated a discrepancy between consistently applying SDM compared to advising what was considered by paediatricians as the best available treatment option. This is exemplified by the frequently observed discussion about pneumococcal vaccination. Not explicitly deliberating on parents’ possible reservations against pneumococcal vaccination, even though this is a recommended treatment option for SCD patients [31], could have been interpreted by patients/parents as not appreciating their expectations, worries and preferences [18, 32].

Our SDM-Q-9 and SDM-Q-Doc scores showed that patients/parents were slightly more positive than paediatricians regarding their level of involvement and about the paediatricians’ effort to check if they had understood the information provided during consultation. The same goes for being involved by the paediatrician in weighing pros and cons of multiple options against personal values and preferences. The option of not (yet) treating the patient as an alternative to treatment was rarely mentioned during consultations. However, it remains unclear whether the wait-and-see option was discussed in previous consultations. Our results indicate that improving SDM during clinical encounters can be achieved in various ways. First, paediatricians should assure the patient/parent that they will help them become sufficiently informed about treatment options. Second, paediatricians could show more effort in helping patients and parents to deliberate about these different options as well as their own preferences. This improves patients’ ability to weigh risks and benefits of treatment options and, consequently, help them make a more informed choice [33–35]. The *teach back* technique could be useful to verify the patients’ understanding of the information that is provided. And finally, given the central role of the nursing staff in paediatric care for children and families that are affected by SCD, their clinical expertise, communication skills and knowledge about patient and parents’ personal beliefs and values can help improve the level of SDM [36–38].

### Study limitations

The OPTION-5 instrument was developed primarily for dyadic conversations between physician and patient [2, 23, 28]. It has been used in the paediatric setting [30, 39] but has not yet been validated for a triadic decision-making process where both child and parents are included in the conversation with the physician. However, the OPTION-5 instrument appears to be just as valuable in this paediatric setting, as SDM is an overarching principle meant to be applicable in all areas of healthcare. A second limitation may be the small number of participating paediatric haematologists and the fact that the study was performed in only one hospital. Since the results could be different in other paediatric haematology centres, extension of this study to other paediatric centres will be helpful to appreciate the generalisability of the present results.

Third, the SDM-Q-9 and SDM-Q-doc questionnaires have not been validated to be interpreted per item separately. Moreover, the SDM-Q-9 and Q-Doc questionnaires show diverging results, suggesting they address different concepts between the two groups of respondents. Despite the SDM-Q-9 scales’ strong internal consistency [40]**,** it cannot be ruled out that the SDM-Q-9 is sensitive to child and parents’ vulnerability towards authority and/or expert halo bias [41]. Studies show that a patient and/or parent sense of a physicians’ infallible authority can negatively impact the quality and outcome of a decision-making process [41–43]. In addition, SDM-Q-9 scores could also be influenced by the level of knowledge of SDM amongst children and their parents who participated in the study. The same goes for patients’ and parents’ level of health literacy in general. If their knowledge was limited, they may have rated their general satisfaction with the encounter rather than the actual SDM process. And finally given the differences in ethnical and cultural backgrounds between the medical team, patients and their parents, it is unclear if and to what extent these differences may have influenced the results.

## Conclusion

**Fig. 5:**
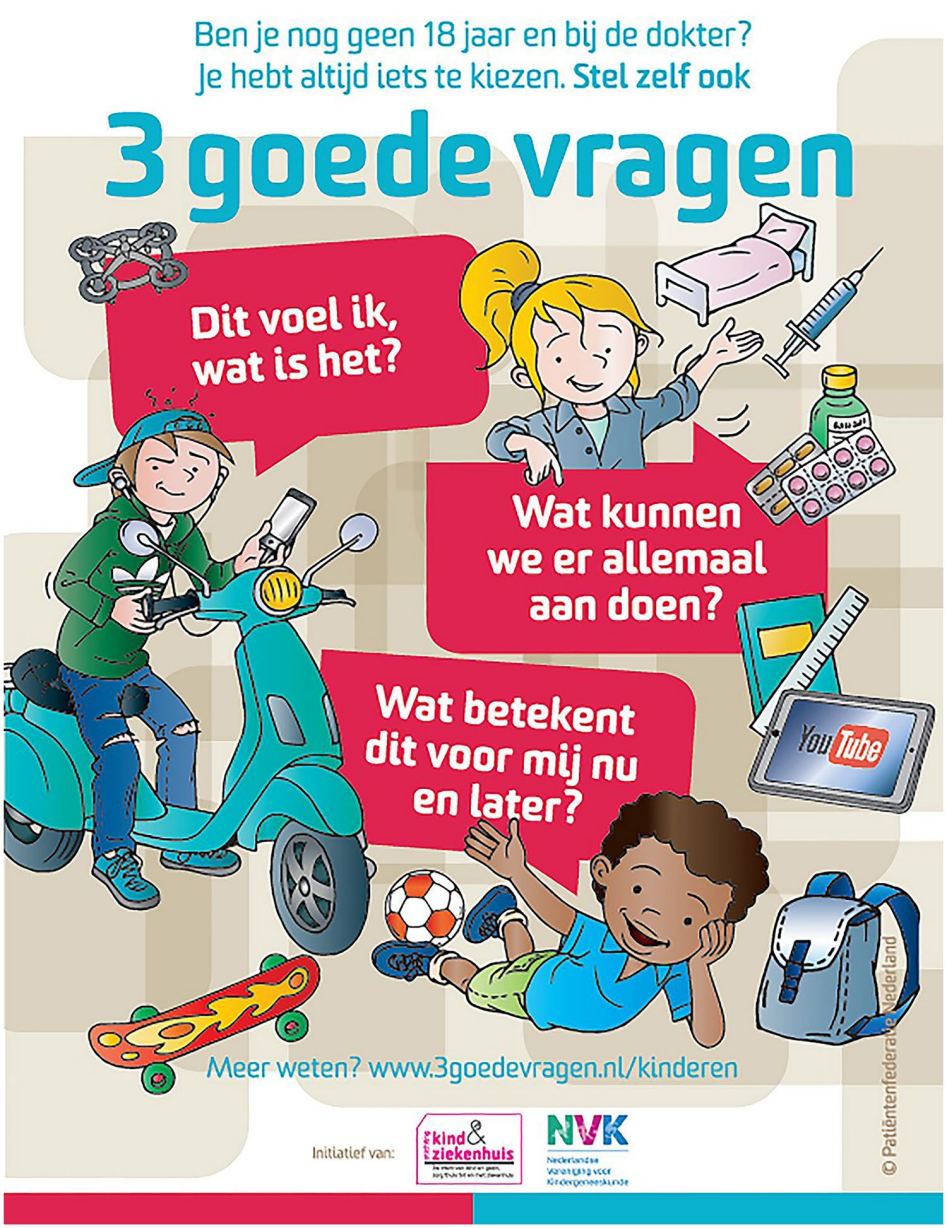
3 Goede Vragen voor Kinderen (3 Good Questions for Children), which can be translated as (1) This is what I feel, what is it?, (2) What can we do about it?, (3) How will this affect me now and later?

The extent to which children with a chronic disorder, such as SCD, and their parents are involved in the decision-making process regarding various treatment options during the course of their disease shows room for improvement. Based on our results, we advocate to promote SDM among children through various tools already available for this purpose, both for children and their patients, such as validated child-centred SDM interventions, such as the *3 Goede Vragen voor Kinderen (3 Good Questions for Children*) (Fig. 5), eHealth smartphone apps and child-centred patient decision aids (PtDAs) [44, 45]. For clinicians, SDM trainings are available with an emphasis on risk communication especially in regard to a population with a different cultural background as the medical team. Third, utilizing nursing staffs’ expertise and communication skills may help facilitate SDM. These three approaches are directly related to the child’s legal and moral right to be involved in all matters concerning its wellbeing and bests interest. Moreover, they help solidify SDM as a method of care that can enhance child participation and improve health outcomes [11, 46, 47]. Future studies will determine if and to what extent better health outcomes and improved quality of child-centred care through SDM can also help decrease health inequities for children with SCD.

## Data Availability

All data generated or analysed during this study are included in this published article and its supplementary information files.
